# Psychological impact of the COVID-19 crisis on young swiss men participating in a cohort study: Differences due to socioeconomic status and work situation

**DOI:** 10.1192/j.eurpsy.2021.293

**Published:** 2021-08-13

**Authors:** S. Marmet, M. Wicki, G. Gmel, C. Gachoud, N. Bertholet, J. Studer

**Affiliations:** 1 Psychiatry, Lausanne University Hospital, Lausanne, Switzerland; 2 Research, Addiction Switzerland, Lausanne, Switzerland; 3 Institute For Mental Health Policy Research, Centre for Addiction and Mental Health, Toronto, Canada; 4 Department Of Health And Social Sciences, University of the West of England, Bristol, United Kingdom

**Keywords:** COVID-19, psychological impact, Switzerland, Socioeconomic status

## Abstract

**Introduction:**

The COVID-19 pandemic impacted daily life worldwide. It may also have had a psychological impact, especially on those with less resources already before the crisis and those who reported substantial changes to their work situation.

**Objectives:**

To investigate whether socioeconomic status before the crisis and changes in work situation during the crisis (unemployment, home-office) are associated with psychological impact in a cohort of young Swiss men.

**Methods:**

A total of 2345 young Swiss men (mean age = 29) completed assessments shortly before (April 2019 to February 2020) and early during the COVID-19 crisis (May to June 2020). Assessments covered psychological outcomes assessed before and during COVID-19 crisis (depression, perceived stress and sleep quality), and assessed during the crisis (fear, isolation and COVID-19 psychological trauma), socioeconomic status (relative financial status and difficulty to pay bills) before the crisis and changes in work situation (unemployment, home-office).

**Results:**

About a fifth of the sample were in partial unemployment or lost their job during COVID-19 crisis. Those in partial or full unemployment, those mostly working from home and those with a lower socioeconomic status already prior to the crisis showed overall higher levels of depression, stress, psychological trauma, fear and isolation.

**Conclusions:**

Even in a country with high social security such as Switzerland, the COVID-19 crisis had a higher psychological impact on those who were already disadvantaged before the crisis or experienced deteriorations in their work situation. Supporting disadvantaged subpopulations during the crisis may help to prevent an amplification of pre-existing inequalities.

**Disclosure:**

No significant relationships.

